# LINKING ETHNIC MINORITY ELDERS TO PROGRAMS AND SERVICES: CHALLENGES TO BUILDING NETWORKS

**DOI:** 10.1093/geroni/igz038.839

**Published:** 2019-11-08

**Authors:** Allen Glicksman, Lauren Ring, Sandra Torres

**Affiliations:** 1 Philadelphia Corporation for Aging, Philadelphia, Pennsylvania, United States; 3 Uppsala Universitet, UPPSALA, Sweden

## Abstract

Building networks that are effective in linking older adults to supportive programs and services often involves challenges related to access, eligibility requirements, the elder's ability to understand enrollment processes, and lack of trust in service providers. For ethnic minority elders these challenges are often greater due to additional linguistic and cultural barriers. The four presentations on this panel address challenges to building effective service networks for ethnic minority elders using data derived from focus groups with members of these communities and those tasked with providing their care. The first presentation (Graham and Tseng) examines the Village model, a model designed to empower older adults, and asks why more Latino, African American and Asian elders do not participate. The second paper (Ågård) looks at communication difficulties as a source for understanding the nature of cross-cultural discussions around end of life issues with ethnic minority patients. The third paper (Ajrouch, Janevic, and Antonucci) explores how caregiving programs for Alzheimer’s Disease patients can be modified to better serve Arabic speaking caregivers. The final paper (Ring, Liebman, Glicksman and Rodriguez) uses data collected among Spanish and Chinese (Mandarin) speaking elders to design a conceptual model which describes how ethnic minority and other elderly navigate the Long Term Care Services and Supports network. Our respondent will place these papers within the growing theoretical work on diversity and care support.

